# Abilifright: A Case Report of Massive Aripiprazole Overdose in a Toddler

**DOI:** 10.5811/cpcem.2021.10.54520

**Published:** 2022-01-28

**Authors:** Nicholus M. Warstadt, Sanjay Mohan, Emma R. Furlano, Jennifer H. Shenker, Eric P. Gibbs, Silas W. Smith

**Affiliations:** * New York University Grossman School of Medicine, Ronald O. Perelman Department of Emergency Medicine, New York, New York; † New York University Grossman School of Medicine, Ronald O. Perelman Department of Emergency Medicine, Division of Medical Toxicology, New York, New York; ‡ New York University Grossman School of Medicine, Department of Pediatrics, New York, New York; § New York University Grossman School of Medicine, Institute for Innovations in Medical Education, New York, New York

**Keywords:** atypical antipsychotic, aripiprazole, overdose, pediatrics, toxicology, emergency medicine

## Abstract

**Introduction:**

Aripiprazole is an atypical antipsychotic with unique receptor-binding properties that has a favorable safety profile in therapeutic doses compared to other antipsychotics. Massive aripiprazole overdose in children, however, presents with profound lethargy and may have neurologic, hemodynamic, and cardiac effects, often requiring admission to a high level of care.

**Case Report:**

We describe a case of a 21-month-old male with a reported 52-milligram aripiprazole ingestion. Initial vital signs were remarkable for tachycardia and hypertension, which rapidly resolved. The patient did not develop hypotension throughout hospitalization. He experienced 60 hours of lethargy. Irritability associated with upper extremity spasms and tremors occurred from 36–72 hours post ingestion, which resolved without intervention. The initial electrocardiogram demonstrated ST-segment depressions in the anteroseptal leads; further cardiac workup was normal. Concurrent medical workup was unrevealing. Aripiprazole and dehydro-aripiprazole serum concentrations sent 46 hours after reported exposure were 266.5 nanograms per milliliter (ng/mL) and 138.6 ng/mL, respectively. He returned to neurologic baseline and was discharged 72 hours after ingestion.

**Conclusion:**

Antipsychotics, including aripiprazole, should be considered as a potential toxicological cause of persistent central nervous system depression; ingestion of a single dose has the potential to cause significant toxicity.

## INTRODUCTION

Aripiprazole has become a commonly prescribed antipsychotic due to its broad indications and tolerability. Unlike other antipsychotics, extrapyramidal symptoms (EPS) and QT interval prolongation are rarely observed with appropriate use. In the few cases of pediatric aripiprazole overdose, prolonged lethargy is common, EPS and hypotension are infrequent, electrocardiographic effects are rare, and no cardiac death has been reported. We describe a case of a 21-month-old male with a large, confirmed aripiprazole overdose complicated by prolonged lethargy, EPS, and possible electrocardiogram (ECG) changes, although baseline ECG was not available for comparison. This report contributes to the understanding that pediatric aripiprazole overdose may present with profound and long-lasting lethargy and EPS. To our knowledge this is the first case report to describe self-limiting spasticity and abnormal ECG findings following confirmed ingestion.

## CASE REPORT

A previously healthy ex-full term, unimmunized, 21-month-old male presented to the pediatric emergency department with lethargy in the setting of an unwitnessed ingestion. Twenty-six two-milligram (mg) aripiprazole tablets were missing from a pill container prescribed for another household member. The ingestion occurred approximately six hours prior to presentation, with subsequent onset of lethargy at the home 2–3 hours later. On presentation, vital signs were as follows: heart rate 151 beats per minute; blood pressure 144/92 millimeters of mercury (mm Hg); respiratory rate 28 breaths per minute; rectal temperature 36.1° Celsius; room air digital oximetry 98%; and weight 12.2 kilograms (kg). Venous blood glucose was 147 mg per deciliter (mg/dL) (normal range 70–99 mg/dL). On initial evaluation, he was lethargic but arousable to physical stimulation. There was no atony, rigidity, tremor, clonus, or spasticity. Bowel sounds were present but decreased. He voided spontaneously. The remainder of the physical examination was unremarkable.

Laboratory analysis, including venous blood gas, complete blood count, electrolytes, hepatic panel, and creatine kinase were normal. Serum acetaminophen, salicylate, ethanol, and troponin concentrations were undetectable. An ECG revealed sinus tachycardia with a heart rate of 160 beats per minute, a QRS interval of 70 milliseconds (ms), and a QTc interval of 359 ms, corrected with Bazett’s formula.^1^ ST-segment depressions were present in the anteroseptal leads (Image). Activated charcoal (AC) was not administered due to sedation and decreased bowel sounds. He was admitted to the pediatric intensive care unit for neurologic and cardiovascular monitoring.

**CPC-EM Capsule**

What do we already know about this clinical entity?

*Aripiprazole overdose in young pediatric patients presents with lethargy. Additional neurologic symptoms and cardiovascular effects are occasionally reported*.

What makes this presentation of disease reportable?

*The relatively large ingestion, prolonged lethargy, and occurrence of extrapyramidal symptoms in this patient add to the understanding of aripiprazole’s toxidrome*.

What is the major learning point?

*Given existing dosing formulations, ingestion of one pill of aripiprazole has the potential to cause severe symptoms in a pediatric patient*.

How might this improve emergency medicine practice?

*This report may help emergency physicians, toxicologists, pediatricians, and intensivists manage pediatric patients with known or suspected aripiprazole overdose*.

The patient’s tachycardia and hypertension resolved within four hours of presentation. The abnormal ECG prompted pediatric cardiology consultation. A transthoracic echocardiogram on hospital day one was normal. QRS and QTc intervals remained within normal limits throughout the hospitalization. The ECG ST-segment depressions persisted at discharge 72 hours after ingestion. Upon discharge, the child was scheduled for a pediatric cardiology clinic appointment to obtain a repeat ECG, but he was lost to follow up.

Decreased level of consciousness persisted for 60 hours post ingestion. He was initially lethargic; then he became more arousable but still slept excessively, before gradually returning to baseline alert state. Starting 36 hours post ingestion, while awake, he was noted to have coarse tremors, worsened with intention, and spasticity of the bilateral upper extremities. While he was asleep, the tremors and spasticity resolved, and he otherwise had normal tone. Pupils were 3 mm, equal and briskly reactive. Hyperreflexia was present in both patellar tendons, without clonus.

Pediatric neurology consultants evaluated other etiologies of the abnormal neurologic exam. Brain non-contrast computed tomography and a 24-hour video electroencephalogram performed on hospital day two were normal. The patient’s tremors and spasticity resolved 72 hours after reported ingestion, and he was at neurologic baseline. Lumbar puncture was deferred due to return to baseline. A social work safety assessment was performed at hospital discharge. Parental education reinforced supervision and safe medication storage.

Aripiprazole and dehydro-aripiprazole serum concentrations obtained approximately 46 hours after reported ingestion (on hospital day two) were 266.5 ng/mL (proposed therapeutic range 150–300 ng/mL)^2^ and 138.6 ng/mL, respectively (ARUP Laboratories, Salt Lake City, UT), confirming aripiprazole exposure.

## DISCUSSION

Aripiprazole is an atypical antipsychotic, differing from other medications in class due to its unique receptor binding properties. Aripiprazole is indicated to treat schizophrenia, bipolar 1 disorder, irritability associated with autism spectrum disorder, Tourette syndrome, and tic disorders in pediatric patients.^3^ First- and second-generation antipsychotics exert much of their effect through dopamine D_2_ receptor antagonism.^4^ In contrast, aripiprazole’s mechanism results from its partial agonist activity at dopamine D_2_ and serotonin 5-HT_1A_ receptors and antagonism at 5-HT_2A_ receptors, with which the drug binds with high affinity.

Aripiprazole has low affinity for alpha1-adrenergic and histamine H_1_ receptors. Aripiprazole elimination mainly occurs through hepatic metabolism involving P450 isozymes CYP2D6 and CYP3A4. The major metabolite, dehydro-aripiprazole, has similar D_2_ receptor affinity. Oral bioavailability is 87%; mean elimination half-lives of aripiprazole and dehydro-aripiprazole are approximately 75 hours and 94 hours, respectively. The mean elimination aripiprazole half-life in CYP2D6 poor metabolizers increases to 146 hours.^3^ Aripiprazole has a high volume of distribution (4.9 liters per kg), indicating extensive extravascular distribution, and greater than 99% of parent drug and its active metabolite are protein bound, particularly albumin. Due to high affinity at central nervous system (CNS) D_2_ and D_3_ receptors, drug dissociation is slow. Toxicokinetics in overdose likely result in prolonged target receptors saturation, as suggested by reports of persistent neurologic sequelae that have followed single, large ingestions.

Neurologic toxicity, particularly lethargy, has been reported as a defining feature in pediatric aripiprazole overdose cases. Several reports describe lethargy in toddlers lasting from 30 hours to seven days.^5^–^9^ Lethargy may be encountered with therapeutic dosing when titration is not performed. One report described a nine-year-old girl with lethargy for 48 hours after starting aripiprazole (15 mg) without dose titration.^10^ In overdose cases, lethargy was self-limited and airway patency and respiratory drive were maintained.

While lethargy is uniformly reported in cases of aripiprazole overdose in younger children, EPS are only occasionally described. Intention tremor was noted in three confirmed aripiprazole ingestions in children younger than three years.^5^–^7^ One case described a 2.5-year-old child with 10-hour serum aripiprazole and dehydro-aripiprazole concentrations of 1420 nanograms per milliliter (ng/mL) and 453 ng/mL, respectively; she exhibited an intention tremor for two weeks.^5^ In all reported cases, tremor resolved without neurological sequelae. Dystonic reactions from aripiprazole occur infrequently. One case described a three-year-old who developed tongue fasciculations, arm twitching, suppressible rhythmic jaw movements, and ataxia following reported ingestion of 200 mg of aripiprazole.^8^ Symptoms improved over three days without intervention. One case reported a six-year-old boy who developed flaccid facial muscles and drooling after the ingestion of aripiprazole 10 mg, which was successfully treated with diphenhydramine 25mg.^9^ In our patient, diphenhydramine administration was deferred due to the profound lethargy that accompanied his EPS.

Cardiac toxicity, particularly QT interval prolongation, is a major concern in antipsychotic drug exposure due to the risk of torsades des pointes, dysrhythmia, and sudden cardiac death. Drug-related QT prolongation typically occurs in a dose-dependent fashion due to impaired currents of the delayed rectifier potassium current channel (I_Kr_), encoded by the human ether-a-go-go related (hERG) gene. In vitro, aripiprazole demonstrates low hERG channel binding, and aripiprazole’s dopamine D_2_ selectivity is approximately 774 times that of I_Kr_.^11^ In a trial of 24 children (mean age, 8.6 years) initiating aripiprazole at therapeutic doses (titrating to a maximum of 15 mg/day), there were no significant changes in QRS or QT intervals from baseline ECGs at the 14-week mark.^12^

Within the scope of our literature review, we found no prior cases of cardiac arrhythmias or death following aripiprazole overdose. Although serum aripiprazole concentrations were not reported, we identified one case report of QRS prolongation lasting nine days in the setting of aripiprazole overdose (400 mg) in a 14-year-old, who was subsequently determined to be a poor metabolizer of CYP2D6.^13^ To our knowledge, our case is the first to describe ST-segment depressions in a patient following aripiprazole overdose. We were unable to infer causality since there was no prior ECG available and his ECG could have been abnormal at baseline; the child was lost to follow-up. Additional cardiac workup, including a transthoracic echocardiogram, was unremarkable; therefore, the significance of the ECG findings is questionable.

Hemodynamic effects of aripiprazole, specifically hypotension, appear less common in both therapeutic dosing and overdose compared to other antipsychotics. While aripiprazole does cause α1-adrenergic receptor antagonism, its affinity for α1-adrenergic receptors is low, and orthostatic hypotension occurred in only 0–1% of children ages 6–18 years old when used therapeutically in clinical trials.^3^ In a literature review of a large case series of 485 children with reported aripiprazole overdose, 5.5–18.8% experienced tachycardia and 0.5% experienced hypotension.^14^ In that study, however, not all patients had confirmed ingestions. Tachycardia and hypotension appear to be more commonly reported in case reports.^5^–^8^ Hypotension has been treated successfully with intravenous fluid bolus administration. To our knowledge, there have been no reported cases of pediatric aripiprazole overdose requiring vasopressors.

Desired therapeutic effects of aripiprazole occur at steady state concentrations of 150–300 ng/mL, with side effects uncommon at a concentration of less than 250 ng/mL.^2^ In therapeutic aripiprazole concentrations between 100–150 ng/mL, striatal and extrastriatal D_2_ and D_3_ receptors remain nearly saturated for up to one week after drug discontinuation.^15^ In this case, an initial serum concentration range of 337.3 – 403.8 ng/mL was calculated assuming instantaneous absorption and using the known 46-hour aripiprazole concentration of 266.5 ng/mL, the above described half-life range, bioavailability, and volume of distribution. This correlates with a calculated initial ingestion dose range of 23.3 – 27.7 mg. Aripiprazole tablets are manufactured in dosages ranging from 2–30 mg. Thus, ingestion of a single pill has the potential to be of significant consequence in young children exhibiting oral exploratory behavior.

## CONCLUSION

In addition to common CNS depressants such as opioids, ethanol, sedative-hypnotics, antihistamines, and antiepileptics, emergency physicians should consider antipsychotics in toxicological etiologies of persistent depressed mental status in children. Aripiprazole should be considered along with other xenobiotics that can cause significant toxicity to young children after ingestion of a single pill or dose. Management of pediatric aripiprazole overdose is largely supportive. Gastric decontamination with activated charcoal may reduce absorption early in presentations,^2^ although safe administration is often precluded by CNS depression. Lethargy following overdose may be profound and last for days. Extrapyramidal symptoms, including tremor or dystonia, have been reported and have been treated with diphenhydramine. Hypotension typically responds to fluid resuscitation. A screening ECG is recommended for infrequent QRS and QT intervals abnormalities. Aripiprazole’s high protein binding and large volume of distribution make hemodialysis unlikely to benefit. As aripiprazole serum concentrations typically do not result within clinically meaningful timeframes, concurrent medical workup to exclude organic etiologies is often required. Families with children should be counseled on potentially significant effects of unintentional overdose at the time of aripiprazole prescription to enact safe storage to mitigate the possibility of unintentional exposure.

## Figures and Tables

**Image:**
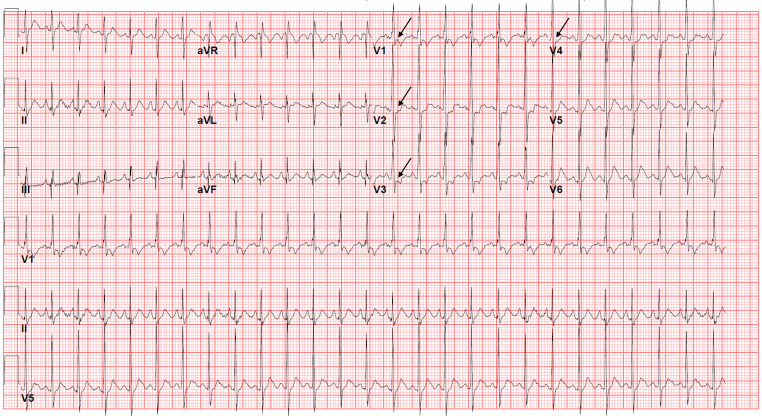
Electrocardiogram shows sinus tachycardia at 160 beats per minute. The QRS complex is 77 milliseconds (ms), and the corrected QT interval is 359 ms by the Bazett method. ST-segment depressions are noted throughout the anteroseptal leads (black arrows).
